# Reirradiation for recurrent glioblastoma: the significance of the residual tumor volume

**DOI:** 10.1007/s11060-025-05042-9

**Published:** 2025-05-01

**Authors:** Sina Mansoorian, Manuel Schmidt, Thomas Weissmann, Daniel Delev, Dieter Henrik Heiland, Roland Coras, Jenny Stritzelberger, Marc Saake, Daniel Höfler, Philipp Schubert, Charlotte Schmitter, Sebastian Lettmaier, Irina Filimonova, Benjamin Frey, Udo S. Gaipl, Luitpold V. Distel, Sabine Semrau, Christoph Bert, Chukwuka Eze, Stephan Schönecker, Claus Belka, Ingmar Blümcke, Michael Uder, Oliver Schnell, Arnd Dörfler, Rainer Fietkau, Florian Putz

**Affiliations:** 1 Department of Radiation Oncology, University Hospital Erlangen, Friedrich-Alexander-Universität Erlangen-Nürnberg, Universitaetsstraße 27, 91054 Erlangen, Germany; 2 Department of Radiation Oncology, University Hospital, Ludwig-Maximilians-Universität München, Munich, Germany; 3 Bavarian Cancer Research Center (BZKF), Munich, Germany; 4 Department of Neuroradiology, University Hospital Erlangen, Friedrich-Alexander-Universität Erlangen-Nürnberg, Erlangen, Germany; 5 Department of Neurosurgery, University Hospital Erlangen, Friedrich-Alexander-Universität Erlangen-Nürnberg, Erlangen, Germany; 6 Department of Neuropathology, University Hospital Erlangen, Friedrich-Alexander-Universität Erlangen-Nürnberg, Erlangen, Germany; 7 Epilepsy Center, Department of Neurology, University Hospital Erlangen, Friedrich-Alexander-Universität Erlangen-Nürnberg, Erlangen, Germany; 8 Institute of Radiology, University Hospital Erlangen, Friedrich-Alexander-Universität Erlangen-Nürnberg, Erlangen, Germany

**Keywords:** Glioblastoma, Recurrence, Chemoradiation, Reirradiation, Tumor volume, Radiotherapy, Prognostic factors

## Abstract

**Purpose:**

Recurrent glioblastoma has a poor prognosis, and its optimal management remains unclear. Reirradiation (re-RT) is a promising treatment option, but long-term outcomes and optimal patient selection criteria are not well established.

**Methods:**

This study analyzed 71 patients with recurrent CNS WHO grade 4, IDHwt glioblastoma (GBM) who underwent re-RT at the University of Erlangen-Nuremberg between January 2009 and June 2019. Imaging follow-ups were conducted every 3 months. Progression-free survival (PFS) was defined using RANO criteria. Outcomes, feasibility, and toxicity of re-RT were evaluated. Contrast-enhancing tumor volume was measured using a deep learning auto-segmentation pipeline with expert validation and jointly evaluated with clinical and molecular-pathologic factors.

**Results:**

Most patients were prescribed conventionally fractionated re-RT (84.5%) with 45 Gy in 1.8 Gy fractions, combined with temozolomide (TMZ, 49.3%) or lomustine (CCNU, 12.7%). Re-RT was completed as planned in 94.4% of patients. After a median follow-up of 73.8 months, 88.7% of patients had died. The median overall survival was 9.6 months, and the median progression-free survival was 5.3 months. Multivariate analysis identified residual contrast-enhancing tumor volume at re-RT (HR 1.040 per cm^3^, p < 0.001) as the single dominant predictor of overall survival.

**Conclusion:**

Conventional fractionated re-RT is a feasible and effective treatment for recurrent high-grade glioma. The significant prognostic impact of residual tumor volume highlights the importance of combining maximum-safe resection with re-RT for improved outcomes.

**Supplementary Information:**

The online version contains supplementary material available at 10.1007/s11060-025-05042-9.

## Introduction

Despite significant advances in the primary treatment of glioblastoma WHO CNS grade 4, relapse in most cases remains inevitable [1]. Managing recurrent glioblastoma is particularly challenging, as no universally accepted standard of care exists. Due to the heterogeneity of recurrent disease, patient-specific factors are crucial in guiding treatment decisions [2]. Key prognostic indicators include prior treatment history and performance status, as consistently highlighted in the literature [3–5].

Treatment options at recurrence often mirror those for primary disease and may include surgery, radiotherapy, systemic therapies (e.g., immunotherapy, targeted agents, angiogenesis inhibitors), and tumor treating fields (TTF). Reoperation, when feasible, is frequently prioritized due to its demonstrated survival advantage over best supportive care [6] and its potential to improve neurological function [7]. However, the role of radiotherapy after re-resection remains poorly defined in the recurrent setting. While re-irradiation (re-RT) is a potential treatment option, randomized studies on its efficacy and safety are limited [5, 8, 9]. Moreover, the timing and selection criteria for re-RT in recurrent glioblastoma are largely unclear.

To address these gaps, this study retrospectively analyzes a cohort of recurrent glioblastoma patients treated with re-irradiation at the University Hospital Erlangen. By evaluating therapeutic outcomes, feasibility, and toxicity, this study aims to provide insights into the role of re-RT and identify patient-specific factors that may influence its success.

## Methods

This retrospective study analyzed 71 patients with glioblastoma IDH wildtype (IDHwt) who underwent re-irradiation (re-RT) between January 2009 and June 2019. Ethical review and approval as well as written informed consent for study participation was not required for this retrospective study in accordance with state legislation (BayKrG Art. 27 (4)) and institutional requirements. This retrospective study followed the 1964 Declaration of Helsinki and its later amendments. All patients provided informed consent for treatment. All patients had a histological diagnosis of glioblastoma, WHO CNS grade 4 according to the 2021 WHO classification [10]. IDH mutation status was determined via immunohistochemistry, while MGMT promotor methylation was assessed using pyrosequencing. Patients with IDH mutation were excluded from the analysis. For primary treatment, all patients underwent maximal safe resection followed by concomitant chemoradiation with temozolomide per the Stupp regimen [1].

Post re-RT, the initial follow-up, including MRI imaging, was conducted 8–12 weeks after treatment. For patients receiving adjuvant or sequential chemotherapy, the first medical appointment occurred approximately 4 weeks after re-RT. Male and female patients aged > 18 years treated for recurrent glioblastoma at the University Hospital Erlangen during the study period were included.

Tumor volumes, including T2-FLAIR hyperintensity and contrast-enhancing regions on 3D T1w sequences, were segmented using a 3D convolutional neural network (DeepMedic) trained on the OpenData-BRATS dataset [11–14]. Both contrast-enhanced T1 and T2-FLAIR sequences were used as inputs. Predictions from the neural network were manually corrected and validated by an expert radiation oncologist using the open-source software 3DSlicer [15].

Tumor volume was calculated as the number of segmented tumor voxels multiplied by the voxel volume [16]. Disease progression was assessed using RANO criteria [17]. Within 12 weeks post-radiochemotherapy, progression was confirmed only if one of the following conditions was met: new contrast enhancement outside the radiation field (beyond the high-dose area or 80% isodose line) or histopathological evidence of a vital tumor. Indicators for histopathological confirmation included more than 70% solid tumor cell nuclei, a significant increase in the MIB-1 proliferation index compared to previous biopsies, evidence of histological progression, or increased anaplasia in tumor cells. Beyond 12 weeks, progression was defined by new contrast-enhancing lesions outside the radiation field with stable or increasing corticosteroid use, a ≥ 25% increase in the sum of tumor diameters compared to prior imaging, or clinical deterioration not attributable to medications or comorbidities. For patients receiving anti-angiogenic therapy, significant increases in T2-FLAIR hyperintensities without T1 contrast enhancement were also considered progression, provided corticosteroid use was stable and other causes (e.g., radiation effects, ischemia, infection) were excluded.

The primary endpoints were feasibility of re-RT, progression-free survival (PFS), and overall survival (OS). PFS was defined as the interval from the start of re-RT to radiologically or pathologically confirmed progression, death, or the last follow-up date. OS was defined as the time from the start of re-RT to death or the last follow-up. Observations were censored at the last follow-up date. Time-to-event outcomes were evaluated using the Kaplan–Meier estimator and the log-rank test. Potential prognostic factors were explored by Cox’s regression analysis. Covariates with a univariate p < 0.20 were included in the multivariate analysis. Optimal prognostic threshold identification was performed using maximally selected rank statistics adjusting log-rank p-values for multiple testing according to Lausen et al. 1994 (R library maxstat) [18, 19]. Identified thresholds were rounded to the nearest integer value. Statistical analyses were performed using IBM SPSS 21 and R 4.3.0 (R Project for Statistical Computing) [20]. P values lower than 0.05 were considered statistically significant.

The location of the glioblastoma recurrence was categorized based on the spatial relationship to the initial radiotherapy field, using isodose lines as reference markers. In-field recurrences were confined to the 95% isodose line, marginal recurrences occurred within the 80%–95% isodose lines, and distant recurrences were located outside the 80% isodose line. Imaging comparisons with initial treatment plans were used to classify the recurrence location.

## Results

This retrospective real-world study analyzed 71 glioblastoma patients with IDH wildtype (IDHwt) who underwent re-irradiation (re-RT) between January 2009 and June 2019 (Table 1). The median follow-up was 73.8 months, with a median age at diagnosis of 59 years (range, 21–77). The cohort comprised 32 women (45.1%) and 39 men (54.9%), all of whom had at least one neurosurgical intervention (biopsy, incomplete resection, or complete resection).

**Table 1:** Characteristics of the reirradiation cohort

Parameter	Whole cohort (N = 71)
Age at re-RT	
Median (range)	59.0 (21.0–77.0)
ECOG at re-RT	
Median (range)	1 (0–4)
Sex, n (%)	
Male	39 (54.9%)
Female	32 (45.1%)
Histological diagnosis at re-RT, n (%)	
Glioblastoma CNS WHO grade 4	71 (100.0%)
IDH1_R132H_ IHC mutation status, n (%)	
IDH1 wildtype	94.4% (67/71)
unavailable	5.6% (4/71)
MGMT promotor hypermethylation, n (%)	
MGMT promotor hypermethylated	52.1% (37/71)
MGMT promotor not hypermethylated	40.8% (29/71)
unavailable	7.0% (5/71)
Contrast-enhancing tumor volume, cm^3^	
Median (range)	11.3 (0.0–65.9)
Mean (IQR)	17.8 (5.9–23.7)
T2-FLAIR hyperintense volume, cm^3^	
Median (range)	93.2 (8.2–256.7)
Mean (IQR)	98.1 (45.1–138.5)
Time of re-RT, n (%)	
First recurrence	61 (85.9%)
Second recurrence	10 (14.1%)
Location of recurrence according to ICD-10, n (%)	
Multiple/Overlapping sites	26 (36.6%)
Temporal	16 (22.5%)
Frontal	15 (21.1%)
Parietal	8 (11.3%)
Occipital	4 (5.6%)
Other supratentorial	2 (2.8%)
Time since primary CRT, months	
Median (range)	15.4 (6.5–49.9)
Location of recurrence, n (%)	
Infield	51 (71.8%)
Distant	11 (15.5%)
Marginal	5 (7.0%)
infield + distant	2 (2.8%)
infield + marginal	1 (1.4%)
marginal + distant	1 (1.4%)
Upfront resection, n (%)	
No upfront resection	40 (56.3%)
Subtotal resection	12 (16.9%)
Complete resection	19 (26.8%)
Administered median single dose,	
Median, Gy (range)	1.80 (1.80-18.0)
Administered median total dose,	
Median, Gy (range)	45.0 (18.0–62.0)
Administered EQD2 α/β = 8, Gy	
Median (range)	44.1 (19.4–62.0)
Prescribed Fractionation scheme, n (%)	
25 × 1.8 Gy (EQD2/8 = 44.1 Gy)	60 (84.5%)
Other:	11 (15.5%)
Concurrent systemic therapy, n (%)	
Temozolomide	35.(49.3%)
CCNU	9 (12.7%)
Bevacizumab Monotherapy	5 (7.0%)
Irinotecan + Bevacizumab	4 (5.6%)
CCNU + Bevacizumab	1 (1.4%)
None	17 (23.9%)
Sequential systemic therapy, n (%)	
Temozolomide	23 (32.4%)
CCNU	8 (11.3%)
Bevacizumab	8 (11.3%)
Irinotecan + Bevacizumab	7 (9.9%)
CCNU + Bevacizumab	5 (7.0%)
None	20 (28.2%)
Feasibility of re-RT, n (%)	
Completed as planned	67 (94.4%)
re-RT aborted	4 (5.6%)

*Re-RT* Reirradiation

MGMT promotor methylation was observed in 37 patients (52.1%), while 29 (40.8%) were unmethylated. MGMT analysis was not feasible in 5 patients (7.0%) due to inadequate tumor material.

Recurrence patterns in relation to the initial radiation field showed that 51 patients (71.8%) had in-field recurrences, 11 (15.5%) had recurrences outside, and 5 (7.0%) had marginal recurrences between the 80–95% isodose lines. Additionally, 2 patients (2.8%) had recurrences both within and outside the radiation field, one (1.4%) had marginal and outside-field recurrences, and one patient (1.4%) had marginal and in-field recurrences. The median contrast-enhancing tumor volume was 11.3 cm^3^ (range, 0–65.9 cm^3^), while the median hyperintense volume on T2-FLAIR was 93.2 cm^3^ (range, 8.2–256.7 cm^3^).

Prior to re-RT, 40 patients (56.3%) did not undergo surgical resection for the recurrence, while 19 (26.8%) underwent complete resection and 12 (16.9%) underwent subtotal resection. Concomitant systemic therapy was received by 54 patients (76.1%), including 10 (14.1%) who received bevacizumab alongside radiotherapy. Sequential systemic therapy was administered to 51 patients (71.8%), with 43 (60.6%) receiving chemotherapy.

The most commonly prescribed re-RT fractionation scheme was 25 × 1.8 Gy up to a total dose of 45 Gy (84.5%, Table 1). Re-RT was completed as planned in 67 patients (94.4%). Early discontinuation of re-RT occurred at 11 × 1.8 Gy, 16 × 1.8 Gy, 22 × 1.8 Gy and 23 × 1.8 Gy corresponding to total doses of 19.8 Gy, 28.8 Gy, 39.6 Gy and 41.4 Gy, respectively. Toxicity was observed in 37 patients (52.1%), though no grade 3 toxicity or larger and no radiation necrosis was observed (Supplemental Table 1).

**Fig. 1:**
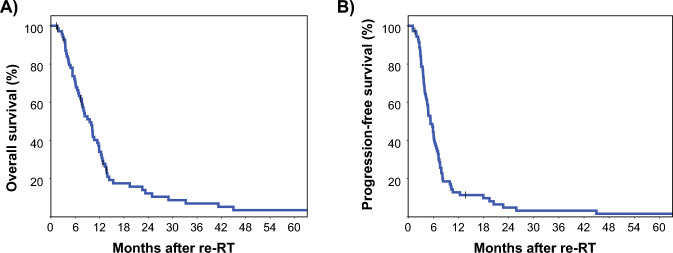
Kaplan–Meier plot of overall and progression-free survival for the re-irradiation cohort (n = 71). Vertical bars represent censored cases

The median progression-free survival (PFS) was 5.3 months (95% CI: 4.1–6.5), with PFS rates at 6 months, 1 year, 2 years, 3 years, and 5 years of 44.3%, 12.9%, 4.9%, 3.3%, and 1.6%, respectively (Fig. 1a). The median overall survival (OS) was 9.6 months (95% CI: 7.2–12.0), with OS rates of 34.1% at 1 year, 12.3% at 2 years, 7.0% at 3 years, and 3.5% at 5 years (Fig. 1b).

Univariate analysis identified recurrence outside the irradiation field (HR 2.457, p = 0.004) as a significant predictor of PFS, while MGMT promotor methylation status was not significant (p = 0.281). In multivariate analysis, the location of the recurrence outside the initial irradiation field remained the single significant factor for PFS (HR 2.488, p = 0.004) (Supplemental Table 2).

**Table 2:** Prognostic factors in univariate and multivariate Cox’s regression analysis for overall survival

Parameter	Univariate		Multivariate	
	HR	p-value	HR	p-value
Total contrast-enhancing tumor volume, cm^3^	1.035	< 0.001	1.040	< 0.001
Sequential chemotherapy	0.485	0.007	0.618	0.123
ECOG, per point	1.290	0.023	1.019	0.902
T2-FLAIR hyperintense volume, cm^3^	1.004	0.032	0.998	0.603
Upfront resection	0.578	0.053	0.670	0.196
Interval since primary CRT, months	0.973	0.055	0.980	0.182
Age, per year	1.018	0.146	1.021	0.130
Sex, female vs. male	1.440	0.156	1.035	0.910
Sequential bevacizumab	0.761	0.344	Not included	
Concurrent bevacizumab	1.312	0.466	Not included	
Out of field recurrence	1.203	0.562	Not included	
MGMT promotor methylation vs. no methylation	1.013	0.961	Not included	
Concurrent chemotherapy	0.997	0.990	Not included	

*CRT* Chemoradiotherapy. Significant covariates are highlighted in bold.

**Fig. 2:**
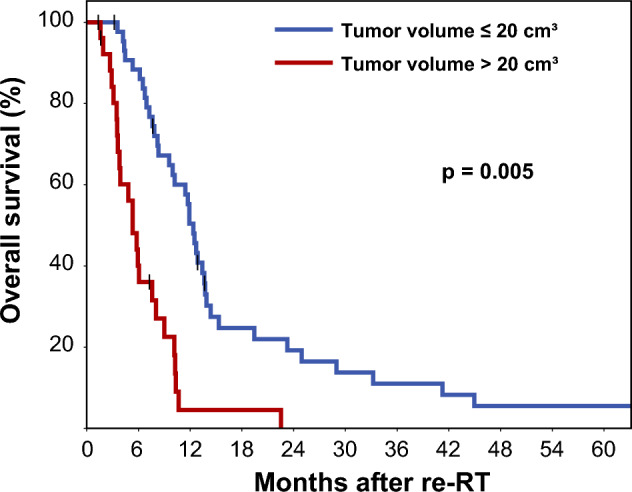
Effect of residual contrast-enhancing tumor at the beginning of re-irradiation on overall survival. The optimal threshold was determined by maximally selected rank statistics. P-value of 0.005 adjusted for multiple testing. Vertical bars represent censored cases

Significant predictors of OS in univariate analysis included ECOG performance index (HR 1.290 per point, p = 0.023), contrast-enhancing tumor volume (HR 1.035 per cm^3^, p < 0.001), T2-FLAIR hyperintense volume (1.004 per cm^3^, p = 0.032), and sequential chemotherapy (HR 0.485, p = 0.007). MGMT promotor methylation status and location of the recurrence in relation to the initial radiation field showed no significant association with overall survival. Multivariate analysis confirmed residual contrast-enhancing tumor volume (HR 1.040 per cm^3^, p < 0.001) as the single dominating prognostic factor (Table 2). A baseline residual tumor volume of 20 cm^3^ at the start of re-RT was identified as optimal threshold for distinguishing patients with favorable and unfavorable prognosis following re-RT (corrected p = 0.005) (Fig. 2).

## Discussion

This retrospective real-world study provides important insights into the outcomes of patients with recurrent CNS WHO grade 4 glioblastoma IDHwt, who underwent re-irradiation. A significant proportion of these patients also received concomitant and sequential systemic therapy. With a median follow-up of 73.8 months, the findings highlight the clinical challenges and key prognostic factors involved in managing this aggressive disease. The median overall survival following re-irradiation was 9.6 months (95% CI: 7.2–12.0), while the median progression-free survival, based on RANO criteria, was 5.3 months (95% CI: 4.1–6.5). These results align with existing literature supporting the role of concomitant radiochemotherapy in recurrent glioblastoma [21–24] (Table 3). However, it has to be noted that older series prior to the 2021 revision of the WHO classification are limited by the inclusion of IDHmut gliomas, which are biologically distinct from IDHwt glioblastoma and respond much more favorable to treatment.

**Table 3:** Overview of published re-irradation series in glioblastoma

Author	Ledermann et al. [46]	Grosu et al. [23]	Combs et al. [47]	Ernst-Stecken et al. [48]	Fokas et al. [27]	Fogh et al. [26]	Minniti et al. [22]	Scholtyssek et al. [39]	Scorsetti et al. [25]	Minniti et al. [49]	Shen et al. [21]	Current work
Toxicity	No high grade toxicity	No G° ≥ 3 toxicity, n = 3 patients with radiation necrosis	No G° ≥ 3 toxicity	No G° ≥ 3 toxicity, 60% G°II symptomatic cerebral edema with need for corticosteroids	No G° ≥ 3 toxicity, no radiation necrosis	N = 1 patient G°3 neurotoxicity (at 40 Gy)	Radiation necrosis 8%	No radiation necrosis, No G° ≥ 3 toxicity	No G° ≥ 3 toxicity	N = 14 patients with G° ≥ 3 hematotoxicity 7% radionecrosis	No G° ≥ 3 toxicity, radiation necrosis 3.4%	No G° ≥ 3 toxicity, no radiation necrosis
Target volume size	GTV 33 cm^3^ (15–150)	GTV 15 cm^3^ (1–61)	PTV 50 cm^3^ (16–149)	GTV 6 cm^3^ (1 -22)	PTV 35 cm^3^ (3–204)	GTV 22 cm^3^ (1–104)	GTV 13 cm^3^ (1–35)	PTV 110 cm^3^	GTV 35 cm^3^	GTV 10 cm^3^ (3 -32)	PTV 202 cm^3^ (20–901)	GTV 11 cm^3^ (0–66)
Positive prognostic factors	GTV < 30 cm^3^	Long interval to primary therapy, planning based on PET examination, chemotherapy	No prognostic factors	No prognostic factors	KPS > 70	Young age, Short therapy interval, Small GTV, RT dose > 35 Gy	MGMT methylation	High KPS, Young age, WHO °III, Female gender, complete resection before re-RT	CRT better than monotherapy	KPS > 70, Long interval to primary therapy, chemotherapy possibly additive effect	Long interval to primary therapy, WHO °III, > 41.4 Gy	Low tumor volume before CRT
mOS (months)	7	8	8	12	9	11	9.7	7.7	9	12.4	9.6	9.6
mPFS (months)	n.a	n.a	5	7	n.a	n.a	5	4.3	8	6	n.a	5.3
Concurrent Chemotherapy	Paclitaxel	TMZ 200 mg/m^2^/d d1-5q28d	TMZ 50 mg/m^2^	In case of Progression Nimustine (ACNU)/ Teniposide (VM-26), PCV, TMZ	TMZ, ACNU/VM-26, PCV	48/147 various chemotherapeutic agents including TMZ, Sunitinib, Sorafenib, BEV + Irino	TMZ 75 mg/m^2^ KOF to RT	TMZ vs. Carboplatin/Etoposid	Fotemustin (75-100 mg/m^2^), Dose dense TMZ (50-100 mg/m^2^) TMZ (75-100 mg/m^2^) 1 week off/on	TMZ daily	TMZ 56%, BEV 14%, TMZ + BEV11%	TMZ 49%, CCNU 13%, BEV 7%, Irino + BEV 6%
Single dose/total dose	6 to 24 Gy	5 to 30 Gy	2 to 36 Gy	7 to 35 Gy	3 (2–5) to 30 Gy (20–60 Gy)	3.5 to 35 Gy	2.5 to 37.5 Gy	2–5 Gy to 36 Gy (30–40)	5 Gy to 25 Gy	5 Gy to 30 Gy	1.5–2 to 41.4 Gy (30–54)	1.8 to 45 Gy
Number of GBM IDHwt	88	35	8	11	53	105	36	53	43	38	87	71
Number of patients	88	44	25	15	53	147	36	64	43	54	118	71

*CRT* Chemoradiotherapy, *TMZ* Temozolomide, *BEV* Bevacizumab, *Irino* Irinotecan, *PCV* Procarbazine, Lomustine, and Vincristine

For instance, a study of 43 patients that included recurrent IDHmut and IDHwt gliomas demonstrated a clear benefit of multimodality therapy, which included surgery and/or stereotactic radiation combined with chemotherapy, compared to chemotherapy alone. Patients in the multimodality therapy group achieved significantly longer progression-free survival (15 vs. 5 months) and overall survival (17 vs. 6 months). Notably, after 2 years, all patients in the chemotherapy-alone group had died, whereas 30% of those in the combination group were still alive, with no increase in toxicity [25].

Evidence further suggests that higher radiation doses exceeding 41.4 Gy are associated with improved outcomes in recurrent disease [21]. In a study of 147 patients with recurrent high-grade gliomas, including 105 glioblastomas (71%), hypofractionated irradiation with a single dose of 3.5 Gy up to a median total dose of 35 Gy showed a trend toward better overall survival with doses above 35 Gy [26]. Studies highlight the importance of optimizing radiation doses and integrating chemotherapy for recurrent glioblastoma and high-grade gliomas. Shen et al. demonstrated that radiation doses above 41.4 Gy were a significant prognostic factor for improved outcomes (HR 0.6) and recommended simultaneous chemoradiation with temozolomide up to 45 Gy for high-grade gliomas [21]. Fokas et al. investigated 53 patients with recurrent glioblastoma treated with re-irradiation (median dose 30 Gy, 3 Gy/fraction) combined with chemotherapy (TMZ, ACNU/VM-26, or PCV). Although chemotherapy provided an additive effect, it did not reach statistical significance [27]. Similarly, Grosu et al. evaluated 44 patients, including 33 with glioblastoma, treated with hypofractionated irradiation (25 Gy in 5 fractions). In the study by Grosu, chemotherapy with temozolomide significantly improved overall survival, with a median OS of 14 months compared to 11 months for radiation alone. Multivariate analysis confirmed the survival benefit of temozolomide [23].

The phase II NRG Oncology/RTOG 1205 trial evaluated 182 patients with recurrent glioblastoma randomized to hypofractionated radiation (35 Gy in 10 fractions) combined with bevacizumab versus bevacizumab alone. The combination therapy significantly improved progression-free survival (7.1 vs. 3.8 months; HR 0.73, p = 0.05) but did not provide an overall survival benefit (10.1 vs. 9.7 months; HR 0.98, p = 0.46) [5]. The feasibility and tolerability of stereotactic radiosurgery for recurrent glioblastoma have been well established in numerous studies [28–30].

Our findings indicate that conventionally fractionated radiotherapy, with or without chemotherapy, is a safe and well-tolerated treatment for recurrent glioblastoma. Planned doses were successfully delivered in 94.4% of patients (67/71), with only four patients receiving 43.1–90.2% of the planned dose. No cases of radionecrosis were observed in the present series. Previous studies using conventional irradiation have reported radionecrosis rates ranging from 0.6% to 10% [21, 24, 31]. According to the QUANTEC review, the risk of symptomatic radiation necrosis following normofractionated radiotherapy (1.8—2 Gy per fraction) is approximately 5% at a cumulative BED (biologically effective dose) of 72 Gy and increases to about 10% at a BED of 90 Gy assuming an α/ß of 2.9 [32]. For example, Combs et al. reported a low incidence of radionecrosis (0.6%) in 172 high-grade glioma patients treated with a mean dose of 36 Gy in 18 fractions [33]. Shen et al. treated 63 patients with a dose of 41.4 Gy in 23 fractions, delivering planned doses in 90% of cases. Radiation necrosis was observed in 3.4% of patients, without symptoms affecting the brainstem or optical system [21]. A substantial fraction of patients in the present series received bevacizumab concurrently or sequentially (Table 1), which together with the conventional fraction may explain the low incidence of radiation necrosis [34, 35].

These results underscore the safety and tolerability of conventionally fractionated radiotherapy for patients with recurrent high-grade gliomas, particularly when adhering to appropriate dosing guidelines.

Numerous studies have found a correlation between age, overall survival, and quality of life in glioblastoma patients [4, 26, 36–40]. In our analysis, increasing age did not emerge as a significant unfavorable predictor of overall survival following re-irradiation. Combs et al. however identified age as a key prognostic factor, with a threshold of 50 years (p < 0.0001) among 233 patients with glioblastomas [38]. Kaul et al. also reported better outcomes in patients younger than 49 years (HR 0.588) [4]. Additionally, Harsh et al. findings emphasized the significant impact of age on survival and quality of life outcomes, with younger patients generally demonstrating a better prognosis [36]. Prior to the revised WHO classification, published series included tumors with IDH-mutation, which are strongly associated with younger age and favorable prognosis [41]. The reduced prognostic significance of age compared to older series can therefore be explained by the strict exclusion of IDH-mutated tumors in the present series.

In our analysis, recurrence outside the irradiation field was a significant multivariate predictor of disease progression (HR 2.49, p = 0.004) but did not significantly correlate with overall survival. This discrepancy may be explained by the availability and feasibility of various local salvage treatments for distant recurrences. The prognostic significance of recurrence location remains underexplored in the literature and warrants further investigation [5].

Contrast-enhancing tumor volume emerged as the dominating predictor of overall survival in our study (HR 1.040 per cm^3^, p < 0.001), with a 1 cm^3^ increase in tumor volume corresponding to a 1.04-fold higher risk of death. A threshold tumor volume of 20 cm^3^ was identified as optimal for distinguishing between good and poor prognosis.

Sharma et al. examined 53 patients with recurrent glioblastoma (75 lesions) treated with stereotactic radiosurgery according to RTOG 95–05 protocols. Tumors ≤ 20 mm received 24 Gy, 21–30 mm received 18 Gy, and 31–40 mm received 15 Gy. Tumor volumes ≥ 15 cm^3^ were associated with worse progression-free survival (HR 2.96) and overall survival (HR 3.78) [42]. Similarly, Elliott et al. found that larger tumor volumes were linked to poorer overall survival (HR 1.23) in 26 recurrent high-grade glioma patients treated with postoperative gamma-knife radiosurgery and temozolomide [43].

Niranjan et al. studied 297 patients with recurrent glioblastoma treated with gamma-knife stereotactic radiosurgery (median dose: 15 Gy). Smaller tumor volumes (< 14 cm^3^) were associated with significantly better survival outcomes, with 1-year survival rates of 42.1% for tumors < 14 cm^3^ and 22.9% for tumors ≥ 14 cm^3^ [44]. These findings support the role of tumor volume as a prognostic factor in recurrent glioblastoma [44, 45].

## Conclusion

This study demonstrates that conventionally fractionated re-irradiation is a safe and feasible treatment option for recurrent CNS WHO grade 4 glioblastoma, with acceptable toxicity and the ability to deliver planned doses in the majority of patients. Residual contrast-enhancing tumor volume at the start of re-RT was the single most important prognostic factor, emphasizing the importance of multimodality treatment approaches in the recurrent setting. The results of this study suggest that recurrent glioblastomas should receive maximum safe resection prior to re-RT to minimize contrast-enhancing tumor volume, if possible. Moreover, re-RT should be initiated early after resection before tumor regrowth has occurred. Glioblastoma patients with low contrast-enhancing tumor volume are optimal candidates for re-RT. A tumor volume threshold of 20 cm^3^ was determined as the optimal cut-off separating patients with favorable and unfavorable re-RT outcome. The study's retrospective design, variability in treatment protocols, and limited molecular data highlight the need for future randomized, multi-center trials to validate these findings and refine patient selection criteria for re-irradiation.

## Supplementary Information

Below is the link to the electronic supplementary material.

Supplementary file1 (DOCX 15 KB)

Supplementary file2 (DOCX 16 KB)

## Data Availability

Raw data supporting the findings of this study are available from the corresponding author (F.P.) upon reasonable request. However, certain portions of the raw data are restricted from sharing due to compliance with data privacy regulations and ethical considerations.
