# Supplementary material for: Reirradiation for recurrent glioblastoma: the significance of the residual tumor volume
Source: J Neurooncol. 2025 May 1;174(1):243–52. doi: 10.1007/s11060-025-05042-9 (PMC12198277; doi:10.1007/s11060-025-05042-9)
Supplement: Supplementary file 1 — Supplementary file1 (DOCX 15 KB) [file 11060_2025_5042_MOESM1_ESM.docx]

Supplemental Table 1: Observed non-hematologic toxicity CTCAE grade 2 or greater

|  | Grade 2 | |
| --- | --- | --- |
| New onset or worsening symptoms | **N** | **Frequency** |
| Fatigue | 12 | 16.9% |
| Dysphasia | 10 | 14.1% |
| Confusion | 7 | 9.9% |
| Dizziness | 6 | 8.5% |
| Amnesia | 3 | 4.2% |
| Nausea | 2 | 2.8% |
| Gait disturbance | 2 | 2.8% |
| Vision decreased | 1 | 1.4% |
| Thromboembolic event | 1 | 1.4% |
| Herpes zoster infection | 1 | 1.4% |

No grade 3 toxicity or greater was observed.
