# Supplementary material for: Reirradiation for recurrent glioblastoma: the significance of the residual tumor volume
Source: J Neurooncol. 2025 May 1;174(1):243–52. doi: 10.1007/s11060-025-05042-9 (PMC12198277; doi:10.1007/s11060-025-05042-9)
Supplement: Supplementary file 2 — Supplementary file2 (DOCX 16 KB) [file 11060_2025_5042_MOESM2_ESM.docx]

Supplemental Table 2: Prognostic factors in univariate and multivariate Cox’s regression analysis for progression-free survival.

| Parameter | Univariate | | Multivariate | |
| --- | --- | --- | --- | --- |
|  | **HR** | **p-value** | **HR** | **p-value** |
| **Out of field recurrence** | **2.457** | **0.004** | **2.488** | **0.004** |
| Sequential bevacizumab | 0.612 | 0.073 | 0.639 | 0.104 |
| Total contrast-enhancing tumor volume, cm³ | 1.011 | 0.155 | 1.014 | 0.070 |
| Age, per year | 1.013 | 0.261 | Not included | |
| MGMT promotor methylation vs. no methylation | 1.325 | 0.281 | Not included | |
| Concurrent chemotherapy | 1.240 | 0.419 | Not included | |
| Sequential chemotherapy | 0.814 | 0.421 | Not included | |
| Concurrent bevacizumab | 1.258 | 0.513 | Not included | |
| Interval since primary CRT, months | 0.993 | 0.606 | Not included | |
| ECOG, per point | 1.055 | 0.671 | Not included | |
| Sex, female vs. male | 1.089 | 0.726 | Not included | |
| T2-FLAIR hyperintense volume, cm³ | 1.000 | 0.963 | Not included | |
| Upfront resection | 0.993 | 0.980 | Not included | |

CRT: Chemoradiotherapy. Significant covariates are highlighted in bold.
